# Dietary flavonoid intake and incidence of erectile dysfunction^1^

**DOI:** 10.3945/ajcn.115.122010

**Published:** 2016-01-13

**Authors:** Aedín Cassidy, Mary Franz, Eric B Rimm

**Affiliations:** 2 Department of Nutrition, Norwich Medical School, University of East Anglia, Norwich, United Kingdom; Departments of; 3 Nutrition and; 4 Epidemiology, Harvard T.H. Chan School of Public Health, Boston, MA; and; 5 Channing Division of Network Medicine, Department of Medicine, Brigham and Women’s Hospital; and; 6 Harvard Medical School, Boston, MA

**Keywords:** anthocyanins, erectile dysfunction, flavanones, flavones, flavonoids

## Abstract

**Background:** The predominant etiology for erectile dysfunction (ED) is vascular, but limited data are available on the role of diet. A higher intake of several flavonoids reduces diabetes and cardiovascular disease risk, but no studies have examined associations between flavonoids and erectile function.

**Objective:** This study examined the relation between habitual flavonoid subclass intakes and incidence of ED.

**Design:** We conducted a prospective study among 25,096 men from the Health Professionals Follow-Up Study. Total flavonoid and subclass intakes were calculated from food-frequency questionnaires collected every 4 y. Participants rated their erectile function in 2000 (with historical reporting from 1986) and again in 2004 and 2008.

**Results:** During 10 y of follow-up, 35.6% reported incident ED. After multivariate adjustment, including classic cardiovascular disease risk factors, several subclasses were associated with reduced ED incidence, specifically flavones (RR = 0.91; 95% CI: 0.85, 0.97; *P*-trend = 0.006), flavanones (RR = 0.89; 95% CI: 0.83, 0.95; *P*-trend = 0.0009), and anthocyanins (RR = 0.91; 95% CI: 0.85, 0.98; *P*-trend = 0.002) comparing extreme intakes. The results remained statistically significant after additional adjustment for a composite dietary intake score. In analyses stratified by age, a higher intake of flavanones, anthocyanins, and flavones was significantly associated with a reduction in risk of ED only in men <70 y old and not older men (11–16% reduction in risk; *P*-interaction = 0.002, 0.03, and 0.007 for flavones, flavanones, and anthocyanins, respectively). In food-based analysis, higher total intake of fruit, a major source of anthocyanins and flavanones, was associated with a 14% reduction in risk of ED (RR = 0.86; 95% CI: 0.79, 0.92; *P = *0.002).

**Conclusions:** These data suggest that a higher habitual intake of specific flavonoid-rich foods is associated with reduced ED incidence. Intervention trials are needed to further examine the impact of increasing intakes of commonly consumed flavonoid-rich foods on men’s health.

## INTRODUCTION

Erectile dysfunction (ED)^7^ has considerable impact on the quality of life of middle-aged men and is a significant global health problem with estimates of a 33–52% prevalence (1–3). Although the origins of ED were thought to be psychogenic or neuropathic, evidence now clearly suggests that the predominant etiology is vascular (4–7). Therefore, ED shares risk factors, including hypertension, obesity, and smoking, with cardiovascular disease (CVD) (3, 4, 8–10); occurrence rises with a progressive clustering of risk factors, and ED is an independent predictor of CVD events (8, 11, 12).

Lifestyle modifications that target CVD risk factors will also have the potential to improve ED, with data from a systematic review of the available 6 randomized controlled trials demonstrating beneficial effects of lifestyle interventions on erectile function after follow-up of 2–24 mo (13). Specifically, they showed that a healthy diet, increased physical activity, and statin therapy were important components to improve men’s sexual health. The specific role of dietary change was demonstrated in a 2-y Mediterranean diet trial where a diet rich in whole grains, fruit, vegetables, nuts, legumes, and olive oil was associated with an improvement in erectile function in subjects with metabolic syndrome (14). This intervention also resulted in an improvement in endothelial function and a decrease in C-reactive protein concentrations, which further highlights the shared underlying mechanisms linking diet, inflammation, vascular function, and ED. In men without established atherosclerosis, those with ED also had poorer endothelial function (15).

To date, few studies have examined the potential role of dietary intake, and the limited available epidemiologic and intervention trial data have predominantly focused on adherence to a Mediterranean diet (14, 16). This diet contains high concentrations of flavonoids, bioactive constituents of foods that may explain some of the observed beneficial effects. Growing evidence supports an improvement in endothelial function and blood pressure after increased dietary flavonoid intake (17–23), suggesting that flavonoids might be more likely than other dietary factors to improve erectile function. Flavonoids are present in many plant-based foods/beverages, including fruit, vegetables, tea, herbs, and wine, and exert anti-inflammatory effects, inhibit LDL oxidation and endothelial NADPH oxidase, modulate endothelial nitric oxide (NO) synthase activity, and augment NO status (17, 18, 22, 24–26).

To our knowledge, the association between different dietary flavonoid subclasses present in plant-based foods on ED incidence has not been investigated.

We therefore examined the relation of the 6 main subclasses of flavonoids commonly consumed in the US diet—flavanones, anthocyanins, flavan-3-ols, flavonoid polymers, flavonols, and flavones—with incidence of ED. We hypothesized, on the strength of the available mechanistic and human trial data on vascular function, that a high intake of anthocyanins, flavanones, and flavan-3-ols would be associated with a reduction in the incidence of ED.

## METHODS

### Subjects

The Health Professionals Follow-Up Study (HPFS) is a prospective cohort study that commenced in 1986 with the recruitment of 51,529 middle-aged male dentists, pharmacists, optometrists, osteopath physicians, podiatrists, and veterinarians (aged 40–75 y). Approximately 97% of participants were of white European descent. Questionnaires were mailed to participants at baseline and biennially to update exposure information and to ascertain the self-report of newly diagnosed diseases (94% response rate) (27). The HPFS was approved by the Harvard T.H. Chan School of Public Health Institutional Review Board.

### Outcome assessment

On the 2000 questionnaire, HPFS participants were asked to rate their ability (without treatment) to have and maintain an erection sufficient for intercourse. Each question included a time grid with year/month increments (before 1986, 1986–1989, 1990–1994, 1995 or later, in the past 3 mo) to allow participants to report historically when and if erectile function changed. Participants were again asked to report their current function (without treatment) in 2004 and 2008. Response options on the 5-point scale included very poor, poor, fair, good, and very good. Only 25,096 men who at baseline in 1998 were without prior diagnosis of ED; prostate, bladder, or testicular cancer; or CVD were included in our analyses. Date of diagnosis was defined as the date of return of the 2000 questionnaire, and we censored at first report of ED. Participants were subsequently censored during follow-up if they developed prostate, bladder, or testicular cancer, and we stopped updating dietary information if they developed CVD during follow-up. Reports of poor or very poor erectile function in any of the periods from 2000 to 2008 were considered incident cases of ED.

### Dietary assessment

Dietary intake data collected from HPFS participants in 1986 and subsequently every 4 y through 2010 were used to calculate cumulative flavonoid subclass intakes. A database for assessment of flavonoid subclass intakes was constructed as previously described (19), which was compiled before the release of the phenol-explorer database. Briefly, intakes of individual compounds were calculated as the sum of the consumption frequency of each food multiplied by the content of the specific flavonoid for the specified portion size. We derived intakes of the subclasses commonly consumed in the US diet—specifically, flavanones, anthocyanins, flavan-3-ols (monomers), flavonols, flavones, and polymers/oligomers (including proanthocyanidins, theaflavins, and thearubigins). We did not evaluate isoflavone intakes because concentrations are very low (<3 mg/d) in the habitual US diet (28), and from a meta-analysis of soy isoflavone intervention studies, we know that isoflavone intakes of 65–153 mg/d are required for blood pressure–lowering effects in hypertensive subjects and 50–99 mg/d for effects on endothelial function (29, 30). Cumulative intakes (energy adjusted) were calculated for a given questionnaire cycle by averaging the intake for the current and preceding food-frequency questionnaires (FFQs). The validity and reproducibility of the FFQs have been reported previously, and correlations between major dietary sources of flavonoids (fruit, vegetables, tea, wine) measured by diet records and FFQs were 0.70, 0.50, 0.77, and 0.83 respectively (31, 32). Our FFQ has not been specifically validated for the intake of flavonoid subclasses, but in a recent study, the sum of 7 flavonoid biomarkers measured in 24-h urine samples was correlated with intakes of fruit and vegetables (0.43–0.66) (33), correlations of a similar magnitude to our validation studies.

### Statistical methods

Participants contributed person-time of follow-up from the date of return of the 2000 questionnaire to the date of first report of ED or the end of follow-up (2010). Updating of dietary information ended at the time participants developed cancer (except nonmelanoma skin cancer), coronary artery disease, angina, or stroke (self-reported percutaneous transluminal coronary angioplasty/coronary artery bypass graft or myocardial infarction) during follow-up to avoid bias due to reverse causality (34). We used a left-truncated Cox proportional hazard regression for time-varying covariates, with a counting process data structure and age in months as the time scale, stratifying also on calendar year (35) to estimate the HR for flavonoid subclass intake in relation to risk of ED by using the lowest intake quintile as the referent group. Covariates were updated biennially (36). We controlled for marital status (married, divorced, separated, widowed, or never married), smoking (never, past, or current 1–14, 15–24, ≥25 cigarettes/d), BMI (in kg/m^2^; <24.9, 25–29.9, or ≥30), physical activity (metabolic equivalents/wk in quintiles), alcohol consumption (0, 0.1–4.9, 5–14.9, 15–29.9, or >30 g alcohol/d), use of multivitamin supplements (yes/no), energy intake (kcal/d, in quintiles), history of hypertension (yes/no), history of myocardial infarction (yes/no), history of hypercholesterolemia (yes/no), and history of diabetes (yes/no).

We conducted further analyses in which we also adjusted for caffeine intake, as well as intake of caffeine/methylxanthine-containing beverages (tea and coffee) and the alternative healthy eating index (as an index of overall diet quality and adherence to dietary guidelines). In other models, we examined the impact of the addition of intakes of amino acids involved in vascular function, including arginine, cysteine, and methionine, to the multivariate model.

In sensitivity analyses, we restricted analyses to healthy men who had never had diabetes or CVD. To identify the impact of risk factors that may modify the relation between flavonoid subclass intake and ED, we examined the effect modification among strata, including age, BMI, smoking, physical activity, and statin use. We conducted food-based analyses on the main dietary sources of the flavonoid subclasses that were associated with a reduction in ED incidence to relate our findings to dietary guidelines. We also examined the joint effects of flavonoid subclasses associated with risk of ED (combined intakes of anthocyanins and flavanones but not flavones because most flavone intake is derived from the same dietary sources as flavanones) with other risk factors including smoking and physical activity. All analyses were conducted with SAS software, version 9 (SAS Institute, Inc.). All *P* values were 2-sided.

## RESULTS

During 10 y of follow-up among the 25,096 participants, 35.6% reported incident ED. Baseline characteristics of the participants according to quintiles of cumulative flavanone intake in 2000 are shown in **Table 1**. Men with a higher flavanone intake smoked less, exercised more, and consumed less alcohol. The flavonoid polymer subclass contributed most to total flavonoid intake (mean intake 207 mg/d; range: 68–442 mg/d), whereas intakes ranged from 3.3 to 35.9 mg/d for anthocyanin and 13.6 to 102.5 mg/d for flavanones. After adjusting for potential confounders, including classic CVD risk factors and a range of lifestyle factors, participants in the highest compared with the lowest quintile of several subclasses of flavonoids had a reduction in the incidence of ED, specifically intakes of flavones (RR = 0.91; 95% CI: 0.85, 0.97; *P*-trend = 0.006), flavanones (RR = 0.89; 95% CI: 0.83, 0.95; *P*-trend = 0.0009), and anthocyanins (RR = 0.91; 95% CI: 0.85, 0.98; *P*-trend = 0.002) (**Table 2**). The results remained statistically significant when we added either caffeine intake or intake of caffeine/methylxanthine-containing beverages (tea and coffee) to the multivariate model—specifically, intakes of flavones (addition of caffeine; flavones (RR: 0.91; 95% CI: 0.85, 0.97; *P*-trend = 0.007), flavanones (RR = 0.89; 95% CI: 0.83, 0.96; *P*-trend = 0.0009, anthocyanins (RR = 0.91; 95% CI: 0.85, 0.98; *P*-trend = 0.002), (addition of tea and coffee intake; flavones RR = 0.91; 95% CI: 0.85, 0.97; *P*-trend = 0.008), flavanones (RR = 0.89; 95% CI: 0.83, 0.96; *P*-trend = 0.001, anthocyanins (RR = 0.91; 95% CI: 0.84, 0.97; *P*-trend = 0.002). The results also remained statistically significant when we added alternative healthy eating index to the multivariate model—specifically, intakes of flavones (RR = 0.87; 95% CI: 0.82, 0.94; *P*-trend = 0.0007), flavanones (RR = 0.87; 95% CI: 0.81, 0.94; *P*-trend = 0.0001), and anthocyanins (RR = 0.88; 95% CI: 0.82, 0.95; *P*-trend = 0.0003). The addition of intakes of arginine, cysteine, or methionine either individually or together to our multivariate model did not materially alter the results (data not shown). When we restricted analyses to healthy men (excluding those with diabetes or CVD, *n* = 21,783, 6768 events), a statistically significant inverse association between intakes of flavones (RR = 0.91; 95% CI: 0.84, 0.99, *P*-trend = 0.04), flavanones (RR = 0.90; 95% CI: 0.83, 0.97; *P*-trend = 0.003), and anthocyanins (RR = 0.93; 95% CI: 0.85, 1.01; *P*-trend = 0.008) was still observed.

**TABLE 1:** Characteristics of the men from the Health Professionals Follow-Up Study by quintile of flavanone intake at baseline^1^

	Flavanone intake, mg/d					
	Q1: 9.7 (*n* = 5019)	Q2: 29.8 (*n* = 5020)	Q3: 51.2 (*n* = 5018)	Q4: 74.6 (*n* = 5020)	Q5: 120.2 (*n* = 5019)	*P*-trend
Age, y	61.1 ± 8.2^2^	62.0 ± 8.5	63.0 ± 8.7	63.6 ± 8.8	64.0 ± 8.9	
BMI, kg/m^2^	26.2 ± 3.7	26.1 ± 3.6	26.0 ± 3.6	25.9 ± 3.4	25.9 ± 3.5	<0.0001
Smoking, %						
Never	45	50	53	55	57	<0.0001
Former	47	45	43	42	40	<0.0001
Current	8	5	4	3	3	<0.0001
Physical activity, MET-h/wk	33.2 ± 44.4	35.8 ± 40.2	36.9 ± 39.0	36.2 ± 36.5	37.8 ± 39.2	<0.0001
Family history of MI, %	9	7	8	8	10	<0.0003
History of hypertension, %	18	17	16	17	18	0.95
History of diabetes, %	8	6	5	5	5	<0.0001
History of hypercholesterolemia, %	48	47	47	48	48)	0.37
Multivitamin use, %	57	57	60	62	63 )	<0.0001
Married, %	88	89	89	91	89	<0.0001
Energy intake, kcal/d	1962 ± 630	2080 ± 662	2140 ± 599	1954 ± 540	1837 ± 593	<0.0001
Alcohol, g/d	11.8 ± 16.3	11.7 ± 14.9	11.5 ± 14.0	10.5 ± 12.3	8.7 ± 11.4	<0.0001
Total flavonoids, mg/d	294 ± 184	308 ± 174	322 ± 170	351 ± 173	394 ± 177	<0.0001
Anthocyanins, mg/d	10.5 ± 7.7	12.3 ± 7.9	12.5 ± 7.8	13.6 ± 8.1	14.6 ± 8.5	
Flavonols, mg/d	16.3 ± 7.4	17.3 ± 7.0	17.5 ± 6.9	18.1 ± 7.0	18.7 ± 7.1	<0.0001
Flavones, mg/d	1.5 ± 0.8	1.9 ± 0.9	2.4 ± 0.9	2.9 ± 0.9	3.4 ± 0.9	<0.0001
Flavan-3-ols, mg/d	131.6 ± 151.7	125.2 ± 143.2	125.0 ± 141.6	130.5 ± 144.5	137.3 ± 149.4	<0.01
Polymers, mg/d	192.7 ± 137	194.2 ± 127	195.8 ± 126	205.5 ± 128	218.7 ± 135	<0.0001

1 Values are standardized to the age distribution of the study population. MET, metabolic equivalent task; MI, myocardial infarction; Q, quintile.

2 Mean ± SD (all such values).

**TABLE 2:** Association between dietary flavonoid intake and ED in 25,096 participants from the Health Professionals Follow-Up Study^1^

	Q1	Q2	Q3	Q4	Q5	*P*-trend
Total flavonoids						
Person-years	40,250	43,785	44,317	44,424	42,269	
Cases of ED, *n*	1629	1804	1886	1856	1740	
Age adjusted	1.0 (reference)^2^	0.96 (0.90, 1.03)	0.97 (0.91, 1.04)	0.92 (0.86, 0.98)	0.92 (0.86, 0.98)	0.006
Multivariate model	1.0 (reference)	0.99 (0.92, 1.05)	1.02 (0.95, 1.09)	0.98 (0.91, 1.05)	0.96 (0.89, 1.02)	0.16
Multivariate model and AHEI	1.0 (reference)	0.97 (0.91, 1.04)	1.00 (0.93, 1.07)	0.96 (0.89, 1.03)	0.94 (0.87, 1.01)	0.08
Flavonols						
Person-years	38,856	43,516	44,959	45,489	42,225	
Cases of ED, *n*	1588	1780	1809	1888	1850	
Age adjusted	1.0 (reference)	0.97 (0.91, 1.04)	0.94 (0.88, 1.00)	0.94 (0.88, 1.00)	0.96 (0.90, 1.03)	0.11
Multivariate model	1.0 (reference)	0.98 (0.92, 1.05)	0.95 (0.89, 1.02)	0.96 (0.90, 1.03)	0.97 (0.91, 1.04)	0.63
Multivariate model and AHEI	1.0 (reference)	0.97 (0.90, 1.04)	0.93 (0.87, 1.00)	0.94 (0.87, 1.01)	0.95 (0.88, 1.02)	0.38
Flavones						
Person-years	40,461	43,689	43,415	43,564	43,917	
Cases of ED, *n*	1613	1765	1801	1884	1852	
Age adjusted	1.0 (reference)	0.95 (0.89, 1.02)	0.92 (0.86, 0.98)	0.92 (0.86, 0.98)	0.88 (0.82, 0.94)	<0.0001
Multivariate model	1.0 (reference)	0.96 (0.90, 1.03)	0.94 (0.87, 1.00)	0.95 (0.89, 1.01)	0.91 (0.85, 0.97)	0.006
Multivariate model and AHEI	1.0 (reference)	0.94 (0.88, 1.01)	0.91 (0.85, 0.98)	0.92 (0.86, 0.99)	0.87 (0.82, 0.94)	0.0007
Multivariate model excluding participants with CVD and diabetes	1.0 (reference)	0.94 (0.87, 1.02)	0.92 (0.85, 0.99)	0.93 (0.86, 1.01)	0.91 (0.84, 0.99)	0.04
Flavanones						
Person-years	43,655	45,841	43,931	41,845	39,773	
Cases of ED, *n*	1739	1826	1866	1813	1671	
Age adjusted	1.0 (reference)	0.96 (0.90, 1.03)	0.94 (0.88, 1.01)	0.92 (0.86, 0.98)	0.85 (0.80, 0.91)	<0.0001
Multivariate model	1.0 (reference)	0.98 (0.92, 1.05)	0.96 (0.89, 1.01)	0.95 (0.89, 1.02)	0.89 (0.83, 0.95)	0.0009
Multivariate model and AHEI	1.0 (reference)	0.97 (0.90, 1.03)	0.95 (0.89, 1.01)	0.93 (0.87, 1.00)	0.87 (0.81, 0.94)	0.0001
Multivariate model excluding participants with CVD and diabetes	1.0 (reference)	0.98 (0.91, 1.06)	0.97 (0.90, 1.05)	0.94 (0.87, 1.02)	0.90 (0.83, 0.97)	0.003
Flavan-3-ols						
Person-years	37,470	44,942	46,285	44,279	42,070	
Cases of ED, *n*	1629	1836	1791	1880	1779	
Age adjusted	1.0 (reference)	1.00 (0.93, 1.06)	0.95 (0.89, 1.02)	1.01 (0.94, 1.07)	1.01 (0.94, 1.08)	0.30
Multivariate model	1.0 (reference)	1.03 (0.96, 1.10)	0.98 (0.92, 1.05)	1.04 (0.97, 1.11)	1.04 (0.97, 1.11)	0.25
Multivariate model and AHEI	1.0 (reference)	1.03 (0.96, 1.10)	0.98 (0.91, 1.05)	1.03 (0.96, 1.10)	1.03 (0.96, 1.10)	0.29
Anthocyanins						
Person-years	35,075	41,856	44,734	46,651	46,730	
Cases of ED, *n*	1485	1803	1885	1868	1874	
Age adjusted	1.0 (reference)	0.98 (0.91, 1.05)	0.93 (0.87, 1.00)	0.89 (0.83, 0.95)	0.86 (0.81, 0.92)	<0.0001
Multivariate model	1.0 (reference)	1.00 (0.93, 1.07)	0.97 (0.91, 1.03)	0.92 (0.86, 0.99)	0.91 (0.85, 0.98)	0.002
Multivariate model and AHEI	1.0 (reference)	0.98 (0.92, 1.05)	0.94 (0.88, 1.01)	0.90 (0.84, 0.97)	0.88 (0.82, 0.95)	0.0003
Multivariate model excluding participants with CVD and diabetes	1.0 (reference)	1.02 (0.95, 1.11)	0.98 (0.90, 1.06)	0.92 (0.85, 1.00)	0.93 (0.85, 1.01)	0.008
Polymeric flavonoids						
Person-years	39,326	43,754	45,193	44,362	42,410	
Cases of ED, *n*	1625	1797	1849	1881	1763	
Age adjusted	1.0 (reference)	0.98 (0.91, 1.04)	0.96 (0.89, 1.07)	0.96 (0.90, 1.02)	0.95 (0.88, 1.01)	0.15
Multivariate model	1.0 (reference)	1.00 (0.93, 1.07)	1.00 (0.93, 1.07)	1.02 (0.95, 1.09)	0.99 (0.92, 1.06)	0.75
Multivariate model and AHEI	1.0 (reference)	0.99 (0.92, 1.06)	0.99 (0.92, 1.06)	1.00 (0.93, 1.07)	0.97 (0.91, 1.05)	0.58

1 Multivariate model adjusted for age, physical activity, smoking, BMI, alcohol, energy, marital status, use of multivitamins, history of CVD, history of hypercholesterolemia, history of hypertension, history of diabetes. AHEI, alternative healthy eating index; CVD, cardiovascular disease; ED, erectile dysfunction; Q, quintile.

2 RR; 95% CI in parentheses (all such values).

**FIGURE 1:**
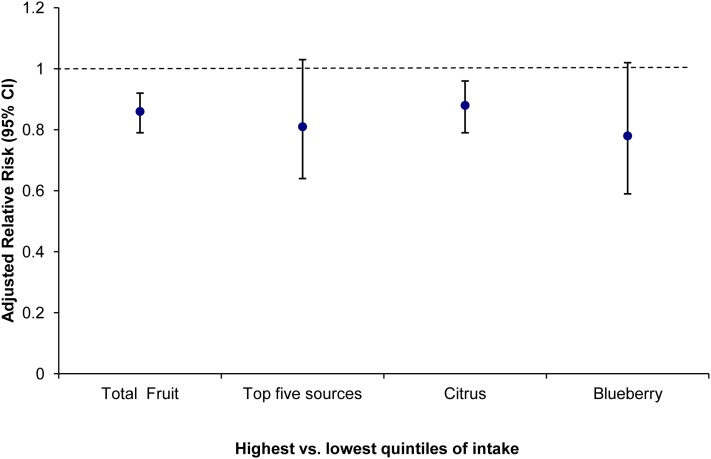
Risk of erectile dysfunction associated with total fruit intake and intake of the top 5 sources of anthocyanins, flavanones, and flavones (strawberries, blueberries, red wine, apples/pears, and citrus products) and intake of citrus and blueberries separately in the Health Professionals Follow-Up Study (*n* = 25,096). RRs were adjusted for the following covariates: marital status, smoking, BMI, physical activity, alcohol consumption, use of multivitamin supplements, energy intake, history of hypertension, history of myocardial infarction, history of hypercholesterolemia, and history of diabetes.

To confirm our findings and to relate the effects to public health and dietary guidelines, we then conducted food-based analyses for total fruit intake and the top 5 sources of anthocyanins, flavones, and flavanones: strawberries, blueberries, red wine, apples/pears, and citrus products. Total fruit intake was associated with a 14% reduction in risk of ED (RR = 0.86; 95% CI: 0.79, 0.92; *P <* 0.0001 comparing quintile 5 with quintile 1), although when we focused on the top sources contributing to the flavonoid subclasses associated with a reduction in ED, we observed a 19% reduction in risk of ED (RR = 0.81; 95% CI: 0.64, 1.03; *P <* 0.008) (**Figure 1**). This inverse association was predominantly due to intake of citrus products (RR = 0.88; 95% CI: 0.80, 0.97; *P = *0.002) and blueberry intake (none compared with >3 portions/wk) (RR = 0.78; 95% CI: 0.59, 1.02; *P = *0.07; Figure 1).

In stratified analyses (**Table 3**), we observed that the inverse association of flavones, flavanones, and anthocyanins with ED was stronger among men aged <70 y compared with those aged ≥70 y (*P*-interaction = 0.002, 0.03, and 0.007 for flavones, flavanones, and anthocyanins, respectively). The inverse association between flavanones and ED was stronger among overweight men compared with those who had a BMI <25 (*P*-interaction = 0.009) (Table 3). We therefore also examined the joint effects of age and BMI, and although we acknowledge that incidence rates were higher in the older men, these analyses suggest that the greatest benefit from an increased intake of flavonones, flavones, and anthocyanins is observed in the younger overweight and obese men; for example, for flavanone intake, comparing extreme quintiles of intakes, the relative risk of ED was 0.85 (95% CI: 0.74, 0.98) for participants aged <70 y with a BMI in the 25–29.9 range and 0.61 (95% CI: 0.47, 0.77) in the BMI ≥30 category. The inverse association was similar in smokers and nonsmokers, across physical activity levels, and by statin use (Table 3). In joint effect analysis, individuals who consumed a high intake of anthocyanins and flavanones and who were physically active had the lowest risk of ED compared with those consuming a low intake of anthocyanins and flavanones and low physical activity levels (RR = 0.79; 95% CI: 0.71, 0.87; *P*-trend < 0.0001).

**TABLE 3:** Associations of anthocyanin, flavanone, and flavone intake with risk of erectile dysfunction across strata of risk factors for participants from the Health Professionals Follow-Up Study^1^

	Q5 vs. Q1								
	Flavones			Flavanones			Anthocyanins		
	RR (95% CI)	*P*-trend	*P*-interaction	RR (95% CI)	*P*-trend	*P*-interaction	RR (95% CI)	*P*-trend	*P*-interaction
Age, y						
<70	0.89 (0.81, 0.98)	0.002	0.002	0.87 (0.79, 0.96)	0.007	0.03	0.84 (0.77, 0.93)	<0.0001	0.007
≥70	1.06 (0.96, 1.16)	0.35		1.01 (0.91, 1.11)	0.99		1.04 (0.94, 1.14)	0.63	
BMI, kg/m^2^									
<25	1.06 (0.94, 1.18)	0.82	0.14	1.01 (0.90, 1.13)	0.99	0.009	1.04 (0.92, 1.16)	0.88	0.48
25–29.9	0.84 (0.76, 0.93)	0.004		0.87 (0.79, 0.96)	0.003		0.83 (0.75, 0.92)	0.0007	
≥30	0.78 (0.65, 0.94)	0.005		0.69 (0.57, 0.83)	0.0002		0.89 (0.74, 1.07)	0.08	
Smoking								
Never	0.86 (0.78, 0.95)	0.005	0.26	0.87 (0.79, 0.96)	0.005	0.73	0.90 (0.81, 1.00)	0.03	0.80
Ever	0.94 (0.85, 1.03)	0.70		0.90 (0.82, 1.00)	0.04		0.91 (0.82, 1.00)	0.01	
Physical activity								
Q1 and Q2	0.94 (0.85, 1.05)	0.20	0.84	0.92 (0.83, 1.03)	0.04	0.55	0.95 (0.85, 1.06)	0.14	0.78
Q3	0.89 (0.80, 1.00)	0.07		0.86 (0.77, 0.96)	0.01		0.89 (0.80, 1.01)	0.03	
Q4 and Q5	0.92 (0.78, 1.08)	0.23		0.93 (0.80, 1.09)	0.68		0.88 (0.75, 1.04)	0.21	
Prevalent hypertension									
No	0.97 (0.90, 1.05)	0.28	0.72	0.94 (0.87, 1.01)	0.06	0.86	0.93 (0.87, 1.01)	0.04	0.80
Yes	0.92 (0.79, 1.07)	0.14		0.91 (0.79, 1.06)	0.25		0.92 (0.79, 1.06)	0.19	
Statin use									
No	0.90 (0.83, 0.98)	0.02	0.71	0.89 (0.82, 0.96)	0.002	0.79	0.92 (0.84, 1.00)	0.01	0.78
Yes	0.90 (0.79, 1.03)	0.13		0.89 (0.78, 1.01)	0.09		0.91 (0.79, 1.04)	0.12	

1 Multivariate model adjusted for age, physical activity, smoking, BMI, alcohol, energy, marital status, use of multivitamins, history of cardiovascular disease, history of hypercholesterolemia, history of hypertension, and history of diabetes. Q, quintile.

## DISCUSSION

To our knowledge, this is the first observational study to suggest that increased habitual intakes of several dietary flavonoids are associated with improved erectile function. Specifically, during 10 y of follow-up and after adjustment for a number of potential confounders, including classic CVD risk factors and a range of lifestyle factors, men in the highest compared with the lowest quintile of intakes of flavanones, flavones, and anthocyanins had a 9–11% reduced incidence of ED. This magnitude of effect is similar to undertaking 7.7–16.5 metabolic equivalent tasks per week of physical activity (37), which equates to approximately 2–5 h of brisk walking per week.

The projected worldwide prevalence of ED for 2025 has been suggested to be >322 million men, with the largest projected increases in the developing world (1). Our data strengthen the knowledge that a healthy diet, specifically one rich in several flavonoids, together with increased physical activity and maintenance of body weight are important components of health to improve sexual health and CVD risk factor reduction. These lifestyle modifications will likely provide benefit regardless of use of current drug therapies, including phosphodiesterase type 5 inhibitors. Although the magnitude of the reduced risk of increased flavonoid intake on erectile function was moderate (9–11%), the potential benefits on improving erectile function could be important at a population level. Interestingly, in our joint analysis, we observed that individuals who consumed a high intake of anthocyanins and flavanones and who were physically active had a 21% lower risk of ED compared with those consuming a low intake of anthocyanins and flavanones and low physical activity levels. In a 2-y intervention trial, in which subjects were advised to achieve 10% weight loss through reduced energy intake and increased physical activity, a 4.0-point improvement in the International Index of Erectile Function (IIEF-5) score was observed (38). These data are supported by prospective data, which found that the 10-y odds of ED was 2.0 comparing men with a BMI ≥28 with men with a BMI <28 at baseline (8), and previous data showed that the RR of ED for obese men (BMI ≥30) was almost twice that of men with an ideal BMI (<25) (RR = 1.9; 95% CI: 1.6, 2.2). Together, these data suggest that even a modest improvement in ED through increased intake of flavonoid-rich foods combined with a moderate increase in physical activity and other lifestyle factor modifications will improve erectile function and offer an opportunity for early intervention for CVD prevention.

In nationally representative data from NHANES, >18 million US men were estimated to have ED, with older men particularly affected (39), and in our cohort, 37.9% reported incident ED over a 10-y period. A 9–11% improvement in ED is important from a public health perspective because ED is an early barometer of poor vascular function and offers a critical opportunity to intervene earlier for the prevention of CVD. The lag time between the onset of ED and CVD presentation is 2–5 y (40–42), and it has been shown to be an independent risk factor for CVD mortality and morbidity (12, 43, 44). Men with ED are likely to be highly motivated to adapt a healthy lifestyle, including dietary approaches, to improve sexual health with resulting benefits to cardiovascular health. In addition, men are likely to recognize dysfunction in their sexual health early, in contrast to risk factors for CVD, which are frequently identified after much of the irreversible vascular damage has occurred.

To date, few studies have specifically examined the potential role of dietary constituents on ED. Recently, a cross-sectional study reported a 39% reduction in risk of ED with higher caffeine intake (intake equivalent to 2–3 cups of coffee/d) (45), although the relative importance of the polyphenol content of coffee was not addressed. The addition of either caffeine intake or caffeine/methylxanthine-containing beverages (tea and coffee) to our model did not attenuate the relation, suggesting that the benefits may be more likely related to the flavonoid content. The only long-term trial observed a beneficial effect of adherence to a Mediterranean diet (14, 46), the data for which are supported by an observational study in men with type 2 diabetes (46). Of the 65 men with metabolic syndrome who were enrolled and followed up for 2 y, a 3-point improvement in the IIEF-5 score was observed (range: 0.6–5.2) in those advised to follow a Mediterranean-style diet (14). Several other trials have evaluated the impact of other lifestyle modifications, including weight loss, exercise, or combined exercise and lifestyle change, and a meta-analysis of the 4 lifestyle modification trials (excluding the 2 pharmacotherapy studies) demonstrated improvements in sexual function. They observed a pooled improvement of 2.4 points in the IIEF-5 score, which is consistent with a relevant improvement in mild ED and a lesser improvement in more advanced ED (13). A clinically important difference in the erectile function domain is accepted to be a 4-point improvement in IIEF-5 score, but the clinical importance of the difference varies substantially according to the degree of severity of ED. For mild ED, a 2.0-point improvement in the IIEF-5 score equates to a clinically important effect, whereas for moderate or severe ED, a respective 5.0- or 7.0-point change in the score is required for a clinical benefit (47). Interestingly, when we added a modified Mediterranean diet score, appropriately adapted for a US habitual diet (alternative healthy eating index), it did not alter our observed inverse associations, providing evidence to suggest the specific importance of high flavonoid intakes.

Penile erection is regulated by a dynamic balance between vasodilator and vasoconstrictor tone, and lack of this homeostasis leads to ED. Vasoconstriction is mediated mostly via RhoA/Rho-kinase pathways, whereas the NO pathway is one of the main vasodilator mediators involved in the functional control of erectile function (48). Available data suggest that several flavonoids can attenuate vascular contraction by inhibiting RhoA/Rho-kinase signaling pathways from experiments using rat aortic rings (49, 50) and population-based studies, and short-term trials support an effect for specific flavonoid subclasses, including flavones, anthocyanins, flavanones, and flavan-3-ols, on vascular function and NO in vivo (18–20, 22–26). Although NO has been accepted as the pathway for mediating the vasorelaxant effects of flavonoids, hydrogen sulfide has been identified as another important physiologic regulator of erectile function via effects on arterial relaxation and (51), and recent in vitro evidence provides evidence to suggest that the hydrogen sulfide pathway was key in mediating the relaxant effect of blueberry juice on rat aortic tissue (52).

Few animal studies have examined the impact of flavonoid ingestion on erectile function, but in one 8-wk study, the flavonol quercetin at high doses (20 and 50 mg · kg^−1^ · d^−1^) ameliorated ED in a diabetic rat model (53); we observed no inverse association for habitual flavonol intake (mean intake 18.4 mg/d). Although habitual flavone intakes were low (mean intake 2.6 mg/d), we did observe a reduction in risk with increased intake, and in an animal model, the flavone apigenin markedly increased NO concentrations and evoked endothelium-dependent vasorelaxation (54). We also hypothesized that increased flavan-3-ol intake would reduce risk, given the wealth of mechanistic support for this subclass, particularly in relation to effects on NO metabolism and endothelial function (17, 20, 21). However, no relation was apparent for habitual intake in this population. Our meta-analysis of flavan-3-ol randomized controlled trials and CVD risk biomarkers suggested that >50 mg epicatechin/d, one of the main flavan-3-ol compounds, is required for beneficial effects on blood pressure (21). By their very nature, FFQs cannot capture all sources of flavonoids, and some sources of flavan-3-ols may not have been accurately captured. For example, dark chocolate, a major source of flavan-3-ols, was not consumed regularly in the 1990s, and most FFQs of that era did not assess different chocolate types. Furthermore, the exact composition of flavan-3-ols in dark and milk chocolate depends on the alkalization process during production (55).

The strengths of this study include the prospective design, the large sample size with long-term follow-up, repeat measures of dietary intake, detailed data on risk factors and confounders for CVD risk, and an up-to-date database of the range of flavonoid subclasses present in the habitual diet. The study also has a number of limitations. Although we adjusted for a range of potential confounders, there is still the possibility of residual or unmeasured confounding from additional unmeasured factors. However, given our detailed and updated adjustment for confounders, it is unlikely these would fully account for the observed findings. Of all the flavonoid classes assessed, only anthocyanins, flavanones, and flavones were associated with ED, suggesting something specific about these subclasses. We used repeated measurements and assessed cumulative intake of diet to more accurately assess long-term flavonoid intake and reduce measurement error. However, flavonoid content varies widely, depending on growing conditions and manufacturing methods, but despite these sources of variation, these data allow us to rank order intakes, which allows comparisons between high and low intakes in large population groups. There are currently a limited range of validated biomarkers to integrate intake with the extensive metabolism these compounds undergo in vivo, so our current knowledge base can only be derived from dietary intake.

Improvement in erectile function in men should therefore be added to the growing list of clinical benefits brought about by a healthy lifestyle, including increased intake of flavonoids. From a public health perspective, ED provides an opportunity for early intervention for the prevention of CVD and represents an opportunity for the early identification of risk factors that are modifiable with lifestyle, particularly in middle-aged men. Our data suggest that increased intake of specific flavonoid-rich foods is associated with a reduced ED incidence, and intervention trials are needed to further examine the potential impact of flavonoids to improve men’s overall health.

## Footnotes

7 Abbreviations used: CVD, cardiovascular disease; ED, erectile dysfunction; FFQ, food-frequency questionnaire; HPFS, Health Professionals Follow-Up Study; IIEF-5, International Index of Erectile Function; NO, nitric oxide.
